# Highlight: The Birds and the Bees and the Bearded Dragons—Evolution of a Sex-Determination System

**DOI:** 10.1093/gbe/evz268

**Published:** 2019-12-19

**Authors:** Casey McGrath

Sex is an ancient and widespread phenomenon, with over 99% of eukaryotes (cells with nuclei) partaking in some form of sexual reproduction, at least occasionally. Given the relative ubiquity and presumed importance of sex, it is perhaps surprising that the mechanisms that determine an individual’s sex vary so spectacularly across organisms. Mechanisms for sex determination can depend on environmental signals, such as temperature, or can be genetically based, with one sex carrying different alleles, genes, or chromosomes—or even different numbers of each of these—from the other. The most well-studied system for sex determination is the XY system, which can be found in most mammals. In this system, females have two of the same type of sex chromosomes (XX) and males have two different types of sex chromosomes (XY).

The existence of multiple sex determination mechanisms across the tree of life implies that these systems experience some degree of turnover, in which one system replaces another. Unfortunately, unraveling evolutionary history of various sex-determination systems can be difficult. In XY systems, the age of the X and Y chromosomes can be estimated by comparing rates of substitution between the two sex chromosomes, since they were once homologs. However, in lineages that have experienced turnover of the sex-determination system, this approach provides no information on whether or how long an XY system may have persisted before replacement with the new system. Luckily, in a new article published in *Genome Biology and Evolution*, titled “Deciphering ancestral sex chromosome turnovers based on analysis of male mutation bias” (Acosta et al. 2019), Professor Diego Cortez and colleagues at the National Autonomous University of Mexico describe a new method for addressing this limitation and providing new insights into the lifespan and turnover of sex-determination systems.

**Fig. 1.**
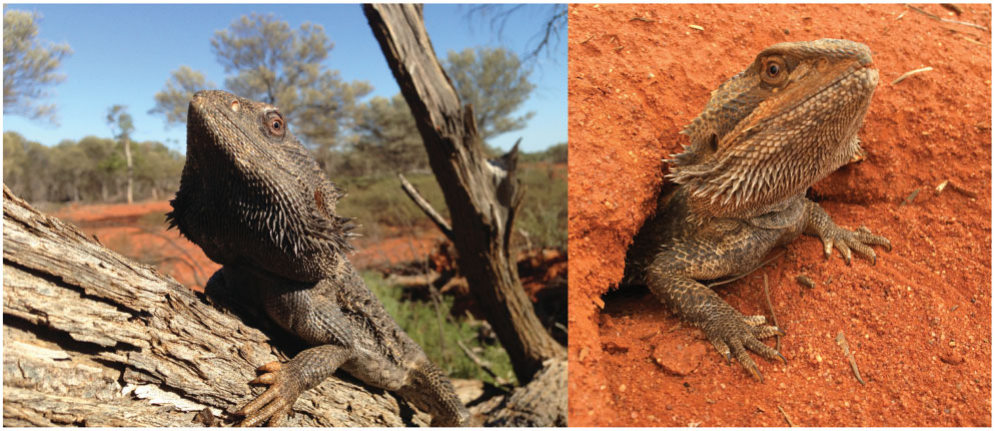
—The acrodont *Pogona*, commonly known as the bearded dragon (Credit: Arthur Georges, University of Canberra).

According to Cortez, development of the new method arose out of necessity: “We were working on the sex chromosomes of the green anole. Some of the analyses suggested that the sex system could be >160 Myr old, that is, it could have originated in the ancestor of pleurodonts (a group including iguanas, anoles, and spiny lizards) and acrodonts (including bearded dragons, shown in fig. 1, and chameleons).” However, because acrodonts do not currently have an XY sex-determination system, Cortez realized that “a method that could corroborate such an evolutionary scenario did not exist.” So, he and his colleagues developed one.

The new analytical method is based on a phenomenon known as male mutation bias. In many vertebrates, male gametes undergo more replication cycles than female gametes, leading to a higher mutation rate on the male-specific (in this case, Y) chromosome and a lower mutation rate on the sex chromosome found more often in females (in this case, X). Male mutation bias has been observed in most mammals, as well as birds, snakes, and fish. Cortez and his colleagues hypothesized that this signature of different mutation rates on ancestral sex chromosomes may persist long after turnover of the sex-determination system and loss of the sex chromosomes themselves.

To verify this, they identified sets of genes that are present on the X chromosome in species with XY sex determination and on autosomes (nonsex chromosomes) in species with alternate sex-determination systems. They then compared the substitution rates at neutrally evolving sites in these genes across lineages—autosomal genes that were once on an X chromosome should have lower rates of evolution than other autosomal genes. In a proof-of-concept experiment, they used this method to analyze substitution rates in placental mammals (which have XY sex determination) and a monotreme, the platypus (which has an alternate system including five X and five Y chromosomes). They found that substitution rates were significantly lower in the X-linked genes from placental mammals than in the autosomal genes from platypus and the nonmammalian vertebrate outgroup species. Based on their simulations, this indicated that the platypus lineage never shared the placental XY system, a finding that is consistent with studies based on other methods.

**Fig. 2.**
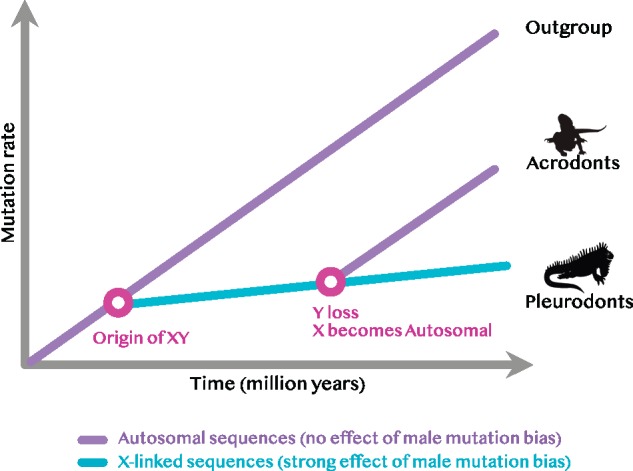
—Sex-determination system evolution, inferred by analysis of the mutation rate, in pleurodonts and acrodonts.

The researchers then applied their method to the analysis of X-linked genes in three pleurodont species (XY sex determination) and their autosomal orthologs in three acrodont species (which have either a variety of sex chromosomes or temperature-dependent sex determination). As with the mammalian analysis, the results showed that the X-linked pleurodont genes had a significantly lower mutation rate than the autosomal genes from the outgroups (in this case, five snake species). Intriguingly, however, the acrodont sequences exhibited substitution rates that were intermediate between those of the X-linked pleurodont genes and the autosomal outgroup genes. This suggested that the acrodonts shared the pleurodont XY system for several million years before it was subsequently lost (fig. 2), as recently as 20–60 Ma in some lineages.

The authors are quick to point out that, while their conclusions are robust, their time estimates “are not super precise” due to the lack of substantial genomic sequences from reptiles. They note that “more sequence data from more reptile species will increase the resolution and validate/correct the observations.”

Despite this constraint, it is clear that this method offers considerable promise for future analyses of sex-determination systems. According to Cortez, their novel method “could be very useful to investigate other lineages where sex chromosome transitions could have happened.” Specifically, the researchers believe that their method could shed light on the rate at which sex chromosomes appear and disappear. They hope that other researchers will test the method in different species and different sex chromosome systems, as it could be used in any system with male mutation bias. Data from other species could ultimately lead to answers about how long sex chromosomes last and whether sex chromosomes have an “expiration date,” thus, providing new insights into the evolutionary processes that drive sex-determination system turnover.
